# Epidemiology of Spinal Cord Injury in British Columbia, Canada: 20 Years of Population-Based Administrative Data

**DOI:** 10.1089/neur.2025.0012

**Published:** 2025-04-09

**Authors:** Michael Bond, Aidan Beresford, Vanessa Noonan, Naama Rotem-Kohavi, Marcel Dvorak, Brian Kwon, Guiping Liu, Jason M. Sutherland

**Affiliations:** ^1^ Centre for Health Services and Policy Research, University of British Columbia, Vancouver, Canada.; ^2^ Praxis Spinal Cord Institute, Vancouver, Canada.; ^3^ International Collaboration on Repair Discoveries (ICORD), University of British Columbia, Vancouver, Canada.; ^4^ Combined Neurosurgery and Orthopaedic Spine Program, University of British Columbia, Vancouver, Canada.

**Keywords:** administrative data, elderly, epidemiology, surgery, trauma, traumatic spinal cord injury, trends

## Abstract

Traumatic spinal cord injury (TSCI) is a debilitating condition that can have significant effects on physical function and overall quality of life. Mechanisms of injury can vary from major trauma to low-energy falls. There has been a recent increase in the number of elderly patients with TSCI. A retrospective analysis of population-based hospital records linked with health care administrative datasets was conducted to measure age-standardized rates of TSCI over time. The study was conducted to describe the epidemiology and demographic characteristics of patients who experienced TSCI between 2001 and 2021 in the province of British Columbia, Canada. Demographic, clinical characteristics, and rates of TSCI were evaluated over time. Linear regression was used to assess changes over time. The study identified 3622 patients with TSCI. The average age at the time of injury was 51.1 (standard deviation [SD] 21.19) and 75.0% were males. The average annual age-standardized rate in this population was 35.4 per million. The overall rate remained stable throughout the study period. The mean age at injury increased from 41.9 to 57.5 over the study period of 2001–2021 (*p* < 0.001). The most frequent causes of injury were low-energy falls (49.9%) and motor vehicle injuries (36.6%). The proportion of injuries related to falls increased over the study period (*p* < 0.001). Motor and sensory complete TSCI were seen in higher rates among younger patients, and cervical spine injuries were most common among all age-groups. The rate of TCSI was consistent during the study period, though the demographic of patients and their injury mechanism changed considerably; elderly low-energy falls were an increasing proportion of cases. Continued vigilance in elderly fall prevention is needed to reduce the incidence of TCSI among the elderly.

## Introduction

Traumatic spinal cord injury (TSCI) is a debilitating condition that can cause permanent motor, sensory, and autonomic dysfunction for those affected.^1–3^ Treatment for TSCIs is complex as many injuries result from an initial traumatic event followed by permanent disability and loss of function.^4^ TSCI further impacts the quality of life for patients after the initial injury with dysregulation in other body systems, such as sexual dysfunction, neuropathic pain, cardiovascular/autonomic instability, and muscular atrophy.^5^ As such, the management of TSCI often requires physician services, rehabilitation, allied health care, and pharmacologic management and treatment to cover a wide range of medical diagnoses, physical disabilities, and mental health conditions.^6–8^ Patients with TSCI have a higher utilization of health services and hospital costs compared to the general population, requiring access to both hospital and community-based services.^9,10^ Treatment and management are challenging and costly to health care systems, society, and the individual, as they absorb privately borne costs that are not reflected in public health spending.^11–13^

In general, TSCIs are rare, with the global annual incidence estimated at 23 persons per million with variations across countries, health care systems, population demographics, and socioeconomic conditions.^14,15^ In Canada, the incidence of TSCI has been estimated to be 32 persons per million based on health care administrative data.^16^ TSCIs are most prevalent among adult males (21–40 years) involved in motor vehicle collisions (MVC)^17,18^ and elderly patients who experience lower energy injuries such as falls from standing height.^19,20^ There is increasing evidence that the incidence of spinal cord injury among the elderly is increasing as populations are aging and individuals are living longer.^21,22^ Elderly patients often have higher medical needs during the acute injury, while undergoing rehabilitation and when discharged back to the community, while younger patients have a longer lifespan after the injury has occurred and have higher health care utilization.^23^ It has been estimated that the average lifetime cost of TSCI in a Canadian population is $336,000 CA per person, with initial hospitalization costs making up a large portion of this cost.^24^ A better understanding of the changes in these demographics of TSCI can allow for better planning and organization of resources to result in better outcomes for patients and improved access to care.^25,26^

To ascertain the most comprehensive rates of TSCI at the population level, administrative data is often used, recording each time a patient accesses health services.^27^ These data, which are often collected for remuneration of health services, cover a range of records including hospital visits and discharges, which merits its applicability for researching TSCI hospital admissions and health care utilization.^28^ Previous work has demonstrated a standard method for estimating TSCI rates using the International Statistical Classification of Disease (ICD) codes to define TSCI and applied in many countries that use this international standard.^29^ While administrative data clarify the epidemiology of TSCI rates, it lacks the depth that clinical data or registries can provide concerning details such as the mechanism of injury, neurological status, and specific treatments initiated.^30,31^ Thus, to better understand the changing epidemiology of TSCI, administrative datasets linked with clinical registry research can provide further insights into TSCI epidemiology.

The primary objective of this study was to report on the epidemiology of TSCI in British Columbia (BC), a province of Canada whose population is approximately five million insured residents. The Canadian single-payer system affords the opportunity to achieve a more comprehensive assessment of the epidemiology of TSCI, as all those who sustain a TSCI within the population are treated within a single-payer system and no patients are treated privately. Here, we utilized population-based health care administrative datasets linked with a clinical spinal cord injury registry to ascertain the annual age-standardized rate of TSCI, demographics, and mechanism of injury. A secondary objective was to evaluate trends over time and investigate if there were changes in the demographics of people experiencing TSCI throughout the study period.

## Materials and Methods

### Study population

This study was based on retrospective data from a population-based observational cohort of people sustaining a TCSI between January 2001 and December 2021. The eligible patient sample represented all incident cases of TSCI over this 20-year period in a geographic population estimated to be more than 5 million.

The TSCI cohort was created based on the ICD-10 diagnosis codes identified in hospital discharge records.^29,31,32^ The cohort identified patients with a new diagnosis and hospitalization for acute cervical, thoracic, and lumbar TSCIs or cauda equina syndrome over the study period. The TSCI case definition was validated by mapping ICD-10 CA codes to International Standards for Neurological Classification of Spinal Cord Injury descriptions of TSCI by level and severity (see Supplementary Appendix for the list of ICD-10 codes).^31^ Ethics approval was obtained from the University of British Columbia Research Ethics Board (REB#: H22-02696).

### Data sources and demographic/clinical variables

The analyses were based on health care administrative data from hospital discharge records, community physician services, and physician billing data that were collected as a routine part of patient care. These records were made available to researchers through Population Data BC.^33^ Population Data BC provides research access to linkable, longitudinal, de-identified patient-level datasets for all publicly insured individuals in the province. Data sources included the Canadian Institute for Health Information Discharge Abstract Database, BC Provincial Vital Statistics Agency (Deaths), the BC Trauma Registry, Medical Services Plan (MSP) Payment Information File, MSP Consolidation File (MSP Registration and Premium Billing), and the Rick Hansen Spinal Cord Injury Registry (RHSCIR), and the protocol for linkage has been described elsewhere.^31,32,34–37^ The demographic and clinical variables collected include age, sex (male and female), Quintile of annual income per person equivalent (QAIPPE), date of admission, date of injury, mechanism of injury (MVC, falls, other), spinal column level (cervical, thoracic, lumbar, and sacral) type/morphology of injury (complete vs. incomplete neurological injury), injury severity score (ISS),^38^ surgery performed (Yes or No), and length of hospital stay (in days). For cohort members, administrative and clinical data were deterministically linked using personal health number and date of birth. Surgical management of TSCI was identified using MSP billing codes for all neurosurgical and orthopedic spinal procedures performed during initial hospital admission. Those without procedure codes during this time were assigned as not receiving surgery.

### Statistical analysis

Initial evaluation of the data was performed for the entire cohort of those with TSCI and then based on age-groups (including <15, 15–24, 25–34, 35–44, 45–54, 55–64, 65–74, and >74), for all demographic and clinical variables, including counts for categorical data and means for continuous data. The crude rate of TSCI was calculated per million using the population of BC during the given year from 2001 to 2021, obtained from provincial population estimates.^39^ Furthermore, age-standardized rates for each individual year and by sex were calculated based on the age population distribution for Canada from the 2001 census reference population.^40^ Trends and relationships over the 21-year study period were examined using analysis of variance statistical methods for continuous variables and chi-square tests for categorical variables. Relationships were examined for age-standardized rates, sex, mechanism of injury, level of neurological impairment, and operative rate. A linear regression model was used to assess trends in age over time for patients with TSCI. All statistical analysis was performed using SAS v9.4, and statistical significance was set at *p* < 0.05.

## Results

During the 21-year study period from 2001 to 2021, we identified 3622 patients with TSCI. The mean age was 51.1 years (standard deviation [SD] 21.19) and 2718 (75.0%) were male. During initial acute hospital admission, 49.4% received spine surgery and the average acute length of stay lasted 67.4 days (SD 107.53). When the rehabilitation hospital admission was included, the average length of stay increased to 101.5 (SD 138.12). In-hospital mortality was found to be 6.2% for all patients (Table 1).

**Table 1.** Demographic and Clinical Data for Patients with TSCI from 2001 to 2021

Variable	
*N*	3622
Age (SD)	51.1 (21.19)
Male	2718 (75.0%)
Charlson Comorbidity Index	
0	2871 (79.3%)
1	398 (11.0%)
2	192 (5.3%)
>3	161 (4.4%)
Quintile of annual income per person equivalent	
1	850 (23.5%)
2	712 (19.7%)
3	675 (18.6%)
4	628 (17.3%)
5	612 (16.9%)
Missing	145 (4.0%)
Injury level	
Cervical (C1–C8)	2519 (69.5%)
Thoracic (T1–T12)	647 (17.9%)
Lumbar (L1–L5)	374 (10.3%)
Sacral /cauda (S1–S5)	60 (1.7%)
Unknown	22 (0.6%)
Complete injury	727 (20.1%)
Incomplete injury	2777 (76.7%)
Unknown	38 (1.0%)
Tetraplegia	2519 (69.5%)
Paraplegia	1081 (29.8%)
Unknown	22 (0.6%)
Spine surgery	
Yes	1789 (49.4%)
No	1833 (50.6%)
Injury Severity Score (ISS) (SD)	24.5 (13.81)
Region	
Rural	573 (15.8%)
Urban	2980 (82.3%)
Unknown	69 (1.9%)
Cause of injury	
Falls	1807 (49.9%)
Motor vehicle collisions	1324 (36.6%)
Other	491 (13.6%)
LOS (acute) in days (SD)	67.4 (107.53)
LOS including acute and rehabilitation in days (SD)	101.5 (138.12)

LOS, length of stay; SD, standard deviation; TSCI, traumatic spinal cord injury.

**FIG. 1.**
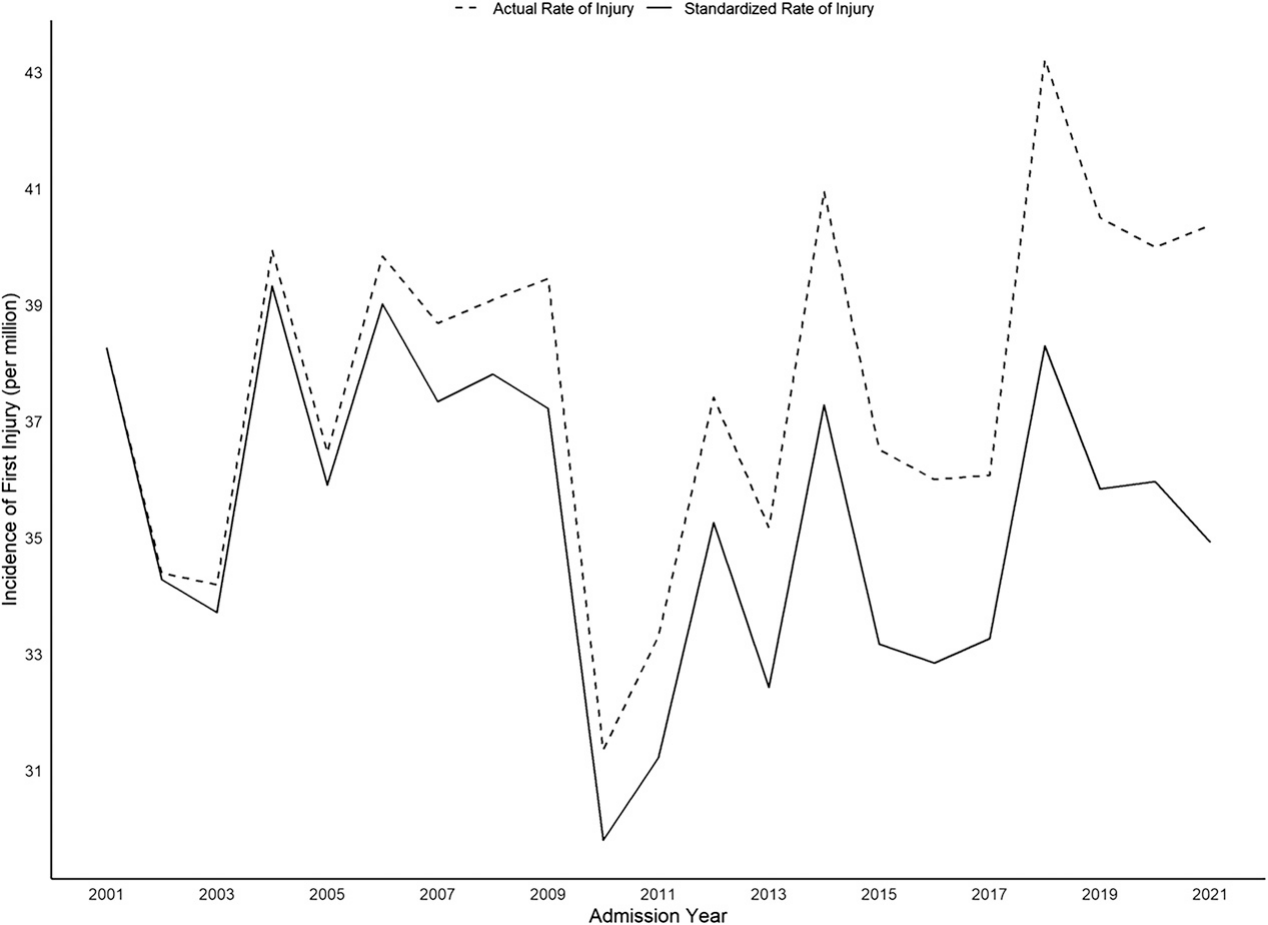
Age-standardized incidence rates of TSCI in British Columbia from 2001to 2021. TSCI, traumatic spinal cord injury.

**FIG. 2.**
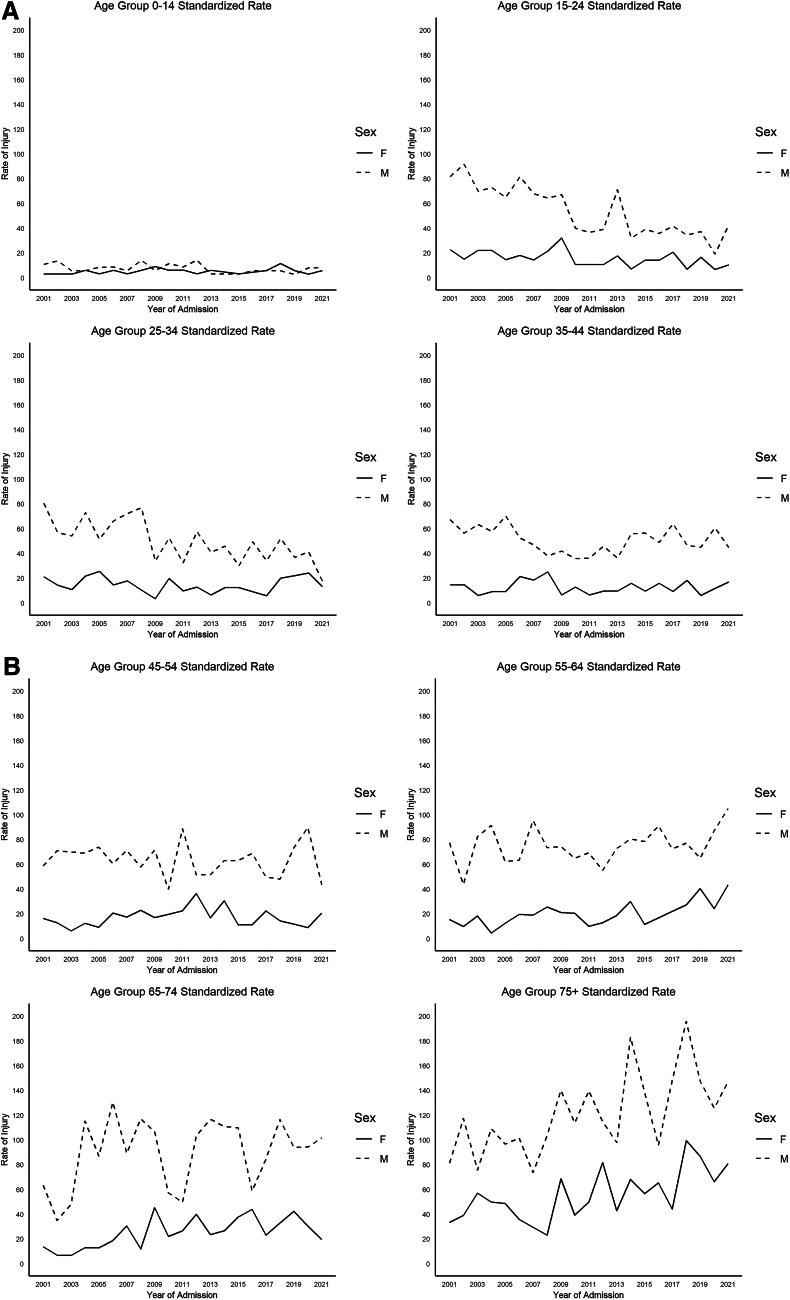
Age-specific rates for TSCI in BC, Canada, by age-group (0–14, 15–24, 25–34, 35–44, 45–54, 55–64, 65–74, and 75 years and older) and sex (male (M) and female (F)) over time (2001–2021). BC, British Columbia.

On average, over the course of the study, the annual age-standardized rate of TSCIs in BC was 35.4 persons per million, and the rate ranged from the lowest 29.8 persons per million in 2010 to the highest 39.3 persons per million in 2004. There was a significant increase in average age, from 41.9 (SD 19.34) to 57.5 (SD 20.66) (*p* < 0.001). Figure 1 presents the age-standardized rates of TSCI by year, and Figure 2A and 2B include the age-group-specific rates over time by sex. There is a notable decrease in rates of TSCI in young males under 35 years and an increase in rates in elderly populations over the age of 65. TSCI occurred most due to falls (49.9%) and MVC (36.6%).

**FIG. 3.**
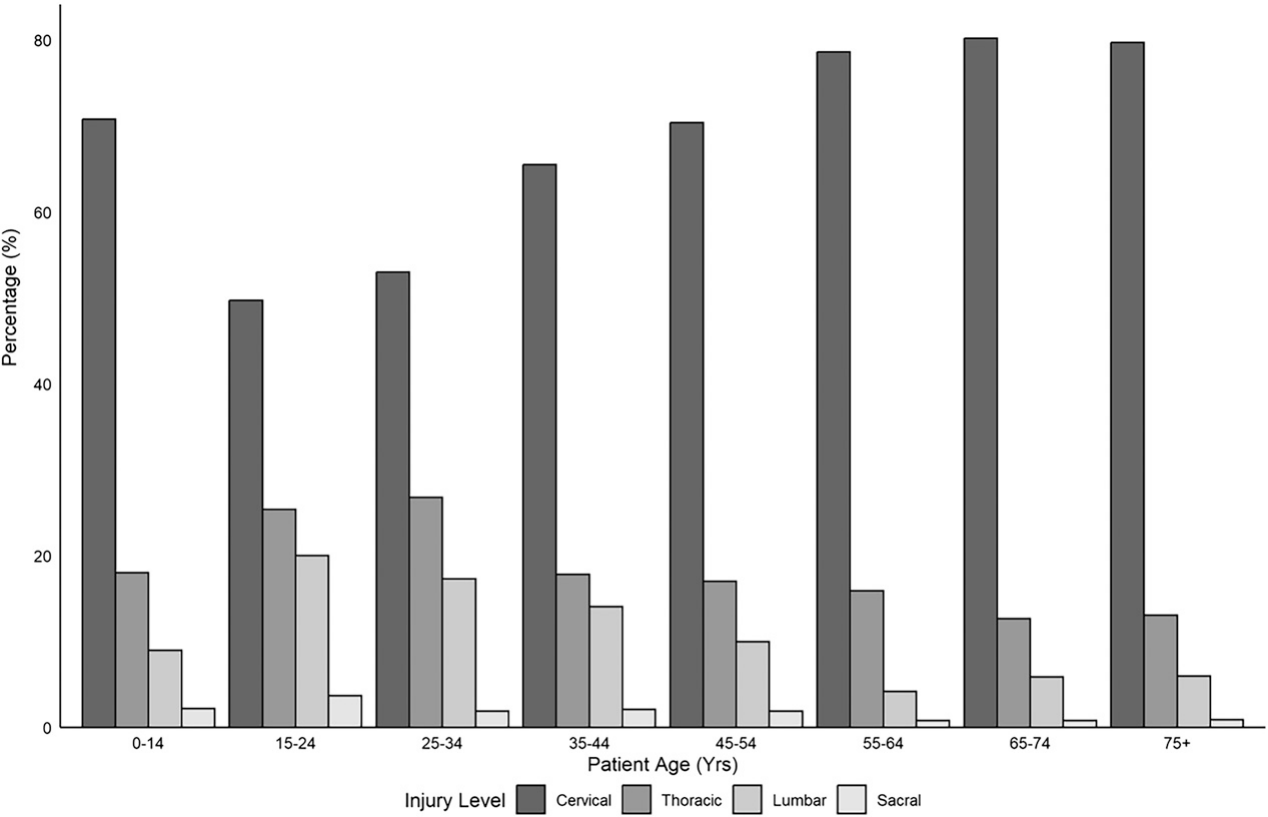
Age-group and level of injury (cervical, thoracic, and lumbar/sacral).

**FIG. 4.**
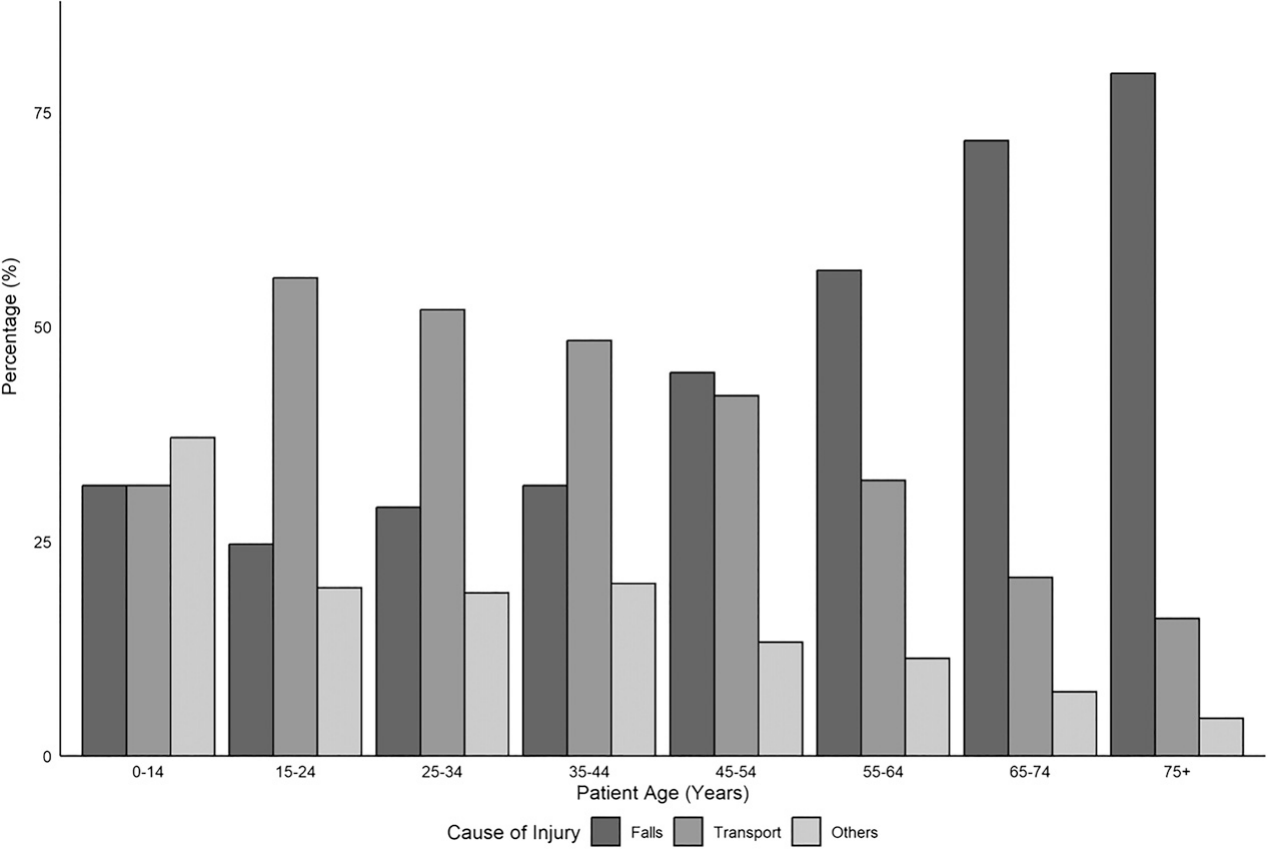
Mechanism of injury by age-group (MVC, falls, and other). MVC, motor vehicle collisions.

**FIG. 5.**
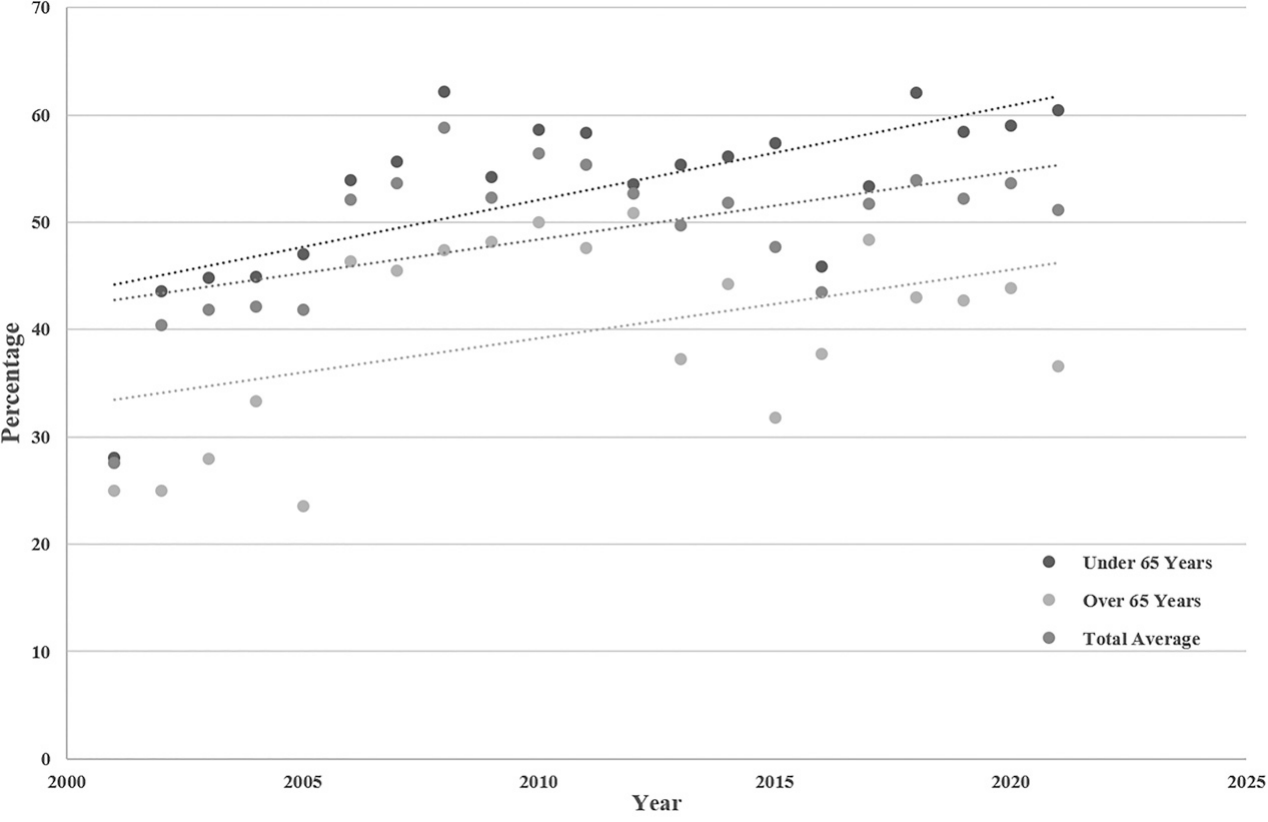
Rate of operative treatment in age-groups 64 and younger versus age 65 and older.

Figure 3 demonstrates the level of injury based on age-groups, with the majority of injuries for all age-groups being in the cervical spine with fewer thoracic and lumbar spine injuries in the older populations (>55 years of age). In total, 729 (20.1%) patients over the study period had complete spinal cord injuries, with higher rates of complete injury in younger age-groups (*p* < 0.001). Most patients had incomplete cervical-level injuries (58.1%), followed by complete cervical and incomplete thoracic injuries (11.0% and 10.2%, respectively). The ISS was calculated with a mean of 26.2 (SD 15.30), considered major trauma,^41^ and decrease in a severity with age (*p* < 0.001). Those who were less than 55 years old were more likely to be involved in MVC, whereas those >55 were more likely to have TSCI because of low-energy falls (see Fig. 4 for details). Figure 5 demonstrates surgical rates which have in total increased over the study period. Breaking down surgery rate by those who were 64 and younger versus 65 and older revealed the older population had less surgeries than the younger population but still increased over time.

## Discussion

Our study found that between 2001 and 2021, the overall average annual rate of TSCI in BC was 35.4 per million patients per year; this rate remained fairly stable during this 21-year period. Over time, the rates of injury increased in those over the age of 75, and the average age of patients presenting with TSCI also increased. The age-specific rates for those over 75 years of age increased from 52.1 per million to 110.4 per million in this patient group during the 20 years of this study period. Furthermore, there was a higher proportion of patients in older age-groups with low-energy mechanisms for TSCI, such as falls, and with incomplete TSCIs. The results demonstrate a shifting demographic amongst those who have TSCI in that a larger proportion falls within an older age-group and further adds to the literature on rates of spinal cord injury within the shifting demographics of a population that is inexorably aging. This work helps to further understand where health care resources for the treatment of TSCI and its aftermath will undoubtedly need to be directed given these changes and highlights the need for fall prevention in the elderly.

The incidence rates for TSCI found through this analysis of more than 20 years of data are similar to other work using national data in Canada that determined the rates of TSCI across the country to be 35.4 TSCIs per million people and an increase in both age and the proportion of patients over the age of 75.^16,42^ Thorogood et al. provided comparative provincial TSCI rates with administrative data in Canada and provided projections of rates up to the year 2019. They found that BC had Canada’s second highest per million rate of TSCI at 36.43 per million (between 2005 and 2016), which is consistent with the rate reported in hospital administrative data.^16^ The rate has remained relatively consistent over time. Prior work evaluating BC from 1995 to 2004 found an average incidence of 35.7 per million and did not notice a significant change over 10 years.^43^

Over the period of the investigation, it was found that the average age at the time of injury for TSCI patients has significantly increased from 41.9 to 57.5 (*p* < 0.001) and that there was an increase in the proportion of those with TSCI who were over the age of 75 years. This trend has been seen in other health care systems, including the United Kingdom, which found that the mean age of TSCI increased by 8.5 years over a 20-year study period, and also in the United States, which found an increase of 9 years over a 50-year study period.^20,44^ This shift can be attributed to an aging population and a larger proportion of elderly patients sustaining SCI from low-energy falls.^43^ The Canadian general population has seen a 12% increase in those aged 85 years and older from 2016 to 2021 and is reportedly set to triple in size by 2046.^45^ Given the results of this and other studies, it is clear to see that the demographics of TSCI are changing with increased rates in elderly populations. This information is valuable to health system planners to ensure that appropriate care and rehabilitation after injury can be provided in a timely, safe, and effective manner.

While overall rates of TSCI have remained relatively stable in the last several decades; however, there was a trend identified for decreased rates of TSCI in young males under 35 years of age. While the primary cause of TSCI in the younger male population continues to be MVC and higher mechanism injuries, there has been much research and success with the improvement and safety of motor vehicles to prevent devastating injuries. This includes research and techniques focused on the use of seat belts, airbags, and reduction in handheld device use while driving, all of which have demonstrated improved safety and reduction in injury.^46–48^ This has been noted in similar research evaluating the incidence of TSCI, with decreasing rates of MVCs over time, providing a potential explanation for these findings.^49,50^

While patients are living longer lives, it is well-established that those who are older are more likely to suffer a low-energy mechanical fall, which can result in a myriad of injuries, including TSCI.^51,52^ Other injuries that have been associated with increasing age from mechanical falls include hip fractures,^53^ hand and wrist injuries,^54^ and vertebral compression fractures,^55^ all of which are associated with increasing morbidity on the individual and burden to the health care system. Given that the elderly population is at increased risk of TSCI from falls, strategies, and policies that support the prevention of low-energy injuries should be developed and implemented. Many strategies have been evaluated for the reduction of falls in the elderly population, including exercise and physical therapy programs to improve balance and strength, modifying the home environment to eliminate tripping hazards, minimizing psychoactive medications, and patient education.^56^ Research should continue in the role of prevention for spinal cord injury with efforts geared toward the elderly population and identifying those who are at risk of TSCI from falls.

This study also determined that the rate of surgical management for patients with acute TSCI across the entire province increased over the study period, with 40.4% of patients receiving surgery in 2002 to 51.2% in 2021. Previous data from British Columbia by Lenehan et al. demonstrated an increase in operation rate from 61.8% in 1995 to 86.4% in 2004, including only data from a large tertiary referral center for TSCI, where often patients are transferred to receive surgical management. Operative management for the majority of TSCI was still the most common treatment method given instability in the spinal column from the injury or ongoing compression requiring spinal instrumentation and decompression techniques. However, in those conditions where there is a spinal cord injury in the absence of instability such as central cord syndrome in elderly patients, nonoperative treatment may be considered. Studies evaluating surgical rates in the elderly populations with TSCI found significantly lower surgical rates than among those who were younger and had higher energy mechanisms.^57^ This is similar to the current study whereby on average surgical rates were lower in those aged 65 years or older, for example in 2021, operation rates were 60.5% in those under 65 and 36.8% in those over 65 years of age. Overall, operative management is still required in most cases of TSCI and is an important part of treatment to prevent further deterioration in neurological status and enable early mobilization and has become more common overtime as the treatment of choice especially in younger patients.

The strength of this study is evident in the use of health care administrative data that documents all interactions with the provincial health care system, providing a broad and in-depth evaluation of TSCI rates for the entire province. However, there are limitations to this study; this study includes only one province in Canada, and thus, the results are not representative of other jurisdictions. However, British Columbia represents a unique province for evaluation of TSCI as it is the third most populous province and has a higher incidence of TSCI than other provinces.^16,58^ Finally, this data was collected and linked with registry data from hospital administrative data-based diagnostic codes entered at the time of discharge or admission for services rendered. These codes may be inaccurately entered and may not represent the true extent of the injury; however, given the care for spinal cord injury, all significant injuries were likely captured, and the validation of the codes used has been previously done by this research group.^31^

## Conclusions

In summary, overall rates of TSCIs have not changed significantly over the last 20 years, but there has been changes in the age specific trends over time. The most noticeable increase is among the elderly population suffering low-energy falls becoming the most common mechanism of TSCIs, further the rates of injury among young male adults are decreasing potentially due to improving safety regulations and trauma prevention. These findings are important to aid in informing health system and policy planning to ensure appropriate infrastructure and programs utilized for TSCI management. Continued efforts to encourage strategies to prevent TSCI should be funded and standardized to ensure implementation nationally, including falls prevention programs and optimizing trauma practices. Future research should evaluate and update changing trends in the TSCI population and explore the potential increased demands on health care placed on the health care system by the changing demographics.

## Transparency, Rigor, and Reproducibility Statement

This study utilized discharge administrative data from Population Data BC for analysis. The data was obtained upon request from 2001 to 2021. Data use agreements prohibit sharing. Access to data provided by the Data Stewards is subject to approval but can be requested for research projects through the Data Stewards or their designated service providers. The following datasets were used in this study: (Consolidation file [includes demographics, registry, and census geodata], Hospital Separations, Medical Services Plan [MSP], Vital Events, and Statistics—Deaths, RHSCIR, BCTR, Vertebase/QISpine). You can find further information regarding these datasets by visiting the PopData project webpage at:

(https://my.popdata.bc.ca/project_listings/15-119/collection_approval_dates).

Due to the study’s retrospective nature, it was not pre-registered nor was analysis. The sample size was determined by the number of patients diagnosed in hospital with TSCI. The study utilized validated ICD-10 code definitions for inclusion. After screening, 3622 patients were included. Investigators knowledgeable in the relevant topic assessed key inclusions and outcomes to report on. Statistical analysis was performed using SAS v9.4. Sample size and power calculations were not conducted, as the dataset obtained was exceptionally large, ensuring the study was adequately powered.

## Abbreviations Used

BC: British Columbia
ICD: International Statistical Classification of Disease
ISS: Injury Severity Score
LOS: Length of Stay
MSP: Medical Services Plan
MVC: Motor Vehicle Collisions
QAIPPE: Quintile of annual income per person equivalent
RHSCIR: Rick Hansen Spinal Cord Injury Registry
SD: Standard Deviation
TSCI: Traumatic Spinal Cord Injury
